# Increased Resistance of Breast, Prostate, and Embryonic Carcinoma Cells against Herpes Simplex Virus in Three-Dimensional Cultures

**DOI:** 10.1155/2013/104913

**Published:** 2013-12-22

**Authors:** Andras Voros, Bernadett Kormos, Tibor Valyi-Nagy, Klara Valyi-Nagy

**Affiliations:** Department of Pathology, College of Medicine, University of Illinois at Chicago, Chicago, IL 60612, USA

## Abstract

In previous studies we found that uveal melanoma cells grown in extracellular matrix (ECM)-containing three-dimensional (3D) cultures have increased resistance against herpes simplex virus type 1 (HSV-1)-mediated destruction relative to cells cultured without ECM. Using additional tumor cell types including MB-231 human breast cancer cells, PC-3 human prostate cancer cells, and P19 mouse embryonal carcinoma cells, we show here that tumor cell lines other than melanoma are also more resistant to HSV-1-mediated destruction in 3D cultures than cells grown in 2D. We also demonstrate here that one mechanism responsible for the increased resistance of tumor cells to HSV-1 infection in 3D cultures is an ECM-mediated inhibition of virus replication following virus entry into cells. These findings confirm and extend previous observations related to the role of the ECM in tumor resistance against HSV-1 and may lead to improved strategies of oncolytic virotherapy.

## 1. Introduction

The oncolytic potential of herpes simplex virus type 1 (HSV-1) has been widely studied both *in vitro* and *in vivo* and genetically engineered HSV-1 strains for tumor therapy are under clinical trials [1–8].

Interestingly, while tumor cells grown in conventional two-dimensional (2D) monolayer cultures are typically quickly killed by HSV-1, virus infection-mediated destruction of tumors *in vivo* is often incomplete [5, 9]. The reasons of increased resistance of tumors against HSV-1 *in vivo* are not well understood but possible mechanisms involved include (i) an impairment of intratumoral virus spread by the extracellular matrix (ECM), (ii) a decreased expression of viral entry receptors, (iii) activation of intracellular tumor defences to viral infection, and (iv) virus clearance by the host immune system [9–18]. It is clear that several of these potential tumor resistance mechanisms are difficult to impossible to study in traditional monolayer tumor cultures.

It is well known that the behaviour of cells is influenced by the ECM and that cancer cells grown in 3D cultures in a polymeric ECM closely mimic many aspects of *in vivo* tumor behavior [19–22]. Numerous data indicate that 3D cultures are more suitable to study key cellular processes, for example, differentiation, proliferation, invasion, and apoptosis, than conventionally used 2D cultures [22–28]. There is evidence that cancer cells grown in 3D culture are more resistant to chemotherapeutic agents and radiation than cells cultured under 2D conditions [27–29]. It is also known that multiple cell types within individual tumors have differential sensitivities to drugs and radiation both *in vivo* and in 3D cultures [27–30]. Based on this knowledge, 3D tumor cultures have been used for preclinical testing of anticancer agents [31–35].

In spite of the known usefulness of 3D cultures for studies of tumor resistance mechanisms, application of 3D culture experimental systems in the field of HSV-1 oncolytic therapy has been quite limited. Earlier work in our laboratory indicated that uveal melanoma cells cultured under 3D condition have increased resistance to HSV-1 compared to 2D cultures [18, 36]. We have shown that in 3D uveal melanoma cultures, morphologically distinct tumor cell populations with increased resistance to HSV-1 are present including tumor cells forming vasculogenic mimicry patterns and multicellular spheroids and individual invasive tumor cells surrounded by ECM [18]. Our observation is that morphologically distinct cell populations present in 3D cultures could be of clinical relevance. For instance, vasculogenic mimicry patterns are present in a wide variety of malignancies including uveal melanomas and their detection is associated with increased mortality [37–39]. Importantly, our previous work with 3D uveal melanoma cultures indicated that the presence of an ECM in 3D tumor cultures was contributing to tumor resistance against HSV-1 by more than one mechanism: the ECM inhibited HSV-1 spread and also mediated inhibition of viral replication following viral entry into tumor cells. Although limited to the use of only one tumor type (uveal melanoma), these observations suggested that virus inoculation of 3D tumor cultures can provide novel insights into mechanisms by which the ECM can modulate tumor resistance against viral oncolytic agents.

To further characterize the role of the ECM in tumor resistance against oncolytic HSV-1 therapy, in the current study we extended our experiments to a variety of additional cell types. Specifically, the aim of the current study was to determine whether it was true for a variety of tumor cell types that (i) cells grown in ECM-containing 3D cultures are more resistant to HSV-1 than cells grown in traditional 2D monolayers without ECM, and that (ii) ECM exposure can mediate inhibition of viral replication following viral entry into tumor cells.

Using four cell lines including MCF10A immortalized nontumorigenic breast epithelial cells, MB231 human breast cancer cells, PC3 human prostate cancer cells, and P19 mouse embryonal carcinoma cells, we show in the current study that tumor cells are more resistant to HSV-1 under 3D conditions than in 2D cultures. We also show that one mechanism responsible for the increased resistance of tumor cells to HSV-1 infection in 3D cultures is an ECM-mediated inhibition of virus replication following virus entry into the cells.

## 2. Materials and Methods

### 2.1. Viruses

Wild-type (wt) HSV-1 strain KOS and recombinant HSV-1 strain K26GFP were amplified and quantitated as described elsewhere [40, 41]. Cells infected with HSV-1 strain K26GFP exhibit punctate nuclear fluorescence at early times in the replication cycle and at later times during infection; a generalized cytoplasmic and nuclear fluorescence, including fluorescence at the cell membranes, can be observed [40]. K26GFP was shown to grow as well as the wild-type virus in cell culture [40].

### 2.2. Cells

MCF-10A (ATCC, CRL-10317) is a nontumorigenic breast epithelial cell line, isolated from a patient with fibrocystic disease. MB-231 (ATCC-HTB-26) human breast cells were derived from the metastasis (pleural effusion) of a breast adenocarcinoma. PC-3 (ATCC CRL-1435) cells originated from a bone metastasis of a prostate adenocarcinoma. MCF-10A, MB-231, and PC-3 cells were grown and maintained in 50/50 Hams/DMEM, DMEM, and F-12 medium, respectively. Mouse embryonal teratocarcinoma P19 cells (ATCC, CRL-1825) were grown in Alpha Minimum Essential Medium with ribonucleosides and deoxyribonucleosides, supplemented with bovine calf serum and fetal bovine serum. Cultures were grown at 37°C in a humidified atmosphere containing 5% CO_2_.

### 2.3. 2D and 3D Cell Cultures

MCF-10A, MB-231, PC-3, and P19 cells were grown on 6-, 12-, or 24-well tissue culture plates in medium either in the presence (3D cultures) or in the absence (2D cultures) of extracellular matrix rich in laminin (Matrigel, BD Biosciences, Bedford, MA). Matrigel is derived from the Engelbreth-Holm-Swarm mouse carcinoma and can be reconstituted as a 3D gel under physiological pH and temperature [42, 43]. For 3D cultures, Matrigel was poured onto tissue culture plates to a depth of approximately 0.2 mm followed by polymerization for 1 hour at 37°C before placement of cells on the Matrigel surface. Cultures were incubated in repeatedly refreshed culture medium for up to 2 weeks and observed daily under an inverted microscope (Leica, Bannockburn, IL).

### 2.4. Determination of Susceptibility of Cells to HSV-1 K26GFP-Mediated Destruction in 2D and 3D Cultures

MCF-10A, MB-231, PC-3, and P19 cells were grown on 6-well tissue culture plates in the presence (3D cultures) or absence of Matrigel (2D cultures). After 3 to 4 days, when all cell types cultured on Matrigel matrix formed their own characteristic three-dimensional structures, tissue culture media were removed and one of the following inocula was gently placed on the surface of the cultures: (i) 0.5 mL of sterile PBS (mock infection); (ii) HSV-1 K26GFP with a calculated MOI of 0.5 PFU per cell diluted in PBS to a final volume of 0.5 mL. After incubation for 1 hour, the original inocula were removed and fresh tissue culture medium (3 mL) was added to each well and further incubated in repeatedly refreshed culture medium for up to 4 weeks. During this 4-week period, cultures were observed daily under an inverted fluorescence microscope (Leica, Bannockburn, IL) for evidence of viral cytopathic effects and GFP expression. The day when at least 95% of the cells were destroyed was noted. Cell death was confirmed by the uptake of the charged cationic dye, Trypan blue. Cells were counted after incubation with Trypan blue (0.2%) for 10 minutes at 37°C and those days were noted and compared when the 95% of the cells died.

### 2.5. Culturing of Previously HSV-1 Inoculated MCF-10A, MB-231, PC-3, and P19 Cells under 2D or 3D Culture Condition

MCF-10A, MB-231, PC-3, and P19 cells were grown on 12-well tissue culture plates in monolayers (2D cultures). When the cultures have reached approximately 70% confluency, culture media were removed and the cells were exposed at 37°C to one of the following inocula: (i) 0.5 mL of sterile PBS (mock infection), or (ii) HSV-1 K26GFP with a calculated MOI of 0.01 PFU per cell diluted in PBS to a final volume of 0.5 mL, or (iii) HSV-1 K26GFP with a calculated MOI of 1 PFU per cell diluted in PBS to a final volume of 0.5 mL. After incubation for 1 hour, the original inocula were removed and the monolayers were washed in sterile PBS twice and 1 mL of sterile PBS was added to each well. Cells were then scraped off using sterile, disposable cell scrapers. Cell solutions were than centrifuged and cell pellets were resuspended in culture medium. Equal volumes (0.25 mL) of the cell solutions were then used to either establish new 2D cultures in 12-well tissue culture plates or 3D cultures as follows. For 3D cultures, Matrigel was poured onto tissue culture plates to a depth of approximately 0.2 mm followed by polymerization for 1 hour at 37°C. Cell suspensions of HSV-1 K26GFP or mock infected MCF-10A, MB-231, PC-3, and P19 cells were mixed with Matrigel 1 : 1 (0.25 : 0.25 mL) and poured on the Matrigel coated wells. Finally, 2 mL of fresh culture medium was added. Both 2D and 3D cultures were then further incubated for 18 hours at 37°C. At 18 hours after the establishment of 2D and 3D cultures, cultures were examined under an inverted fluorescence microscope (Leica, Bannockburn, IL) for evidence of GFP expression and the percentage of GFP-expressing cells was determined by counting the number of GFP positive and GFP negative cells in 16 high power microscopic fields for each studied culture dish.

## 3. Results and Discussion

### 3.1. Different Growth Patterns of MCF-10A, MB-231, PC-3, and P19 Cells Cultured on Matrigel Matrix

To examine the effect of laminin-rich ECM on cell morphology, MCF-10A, MB-231, PC-3, and P19 cells were either seeded onto culture plates coated with a 0.2 mm layer of Matrigel or onto plates without Matrigel. All cell lines formed monolayers (2D cultures) in the absence of Matrigel but the cell lines formed cell line-specific 3D cultures in dishes containing Matrigel.

**Figure 1:**
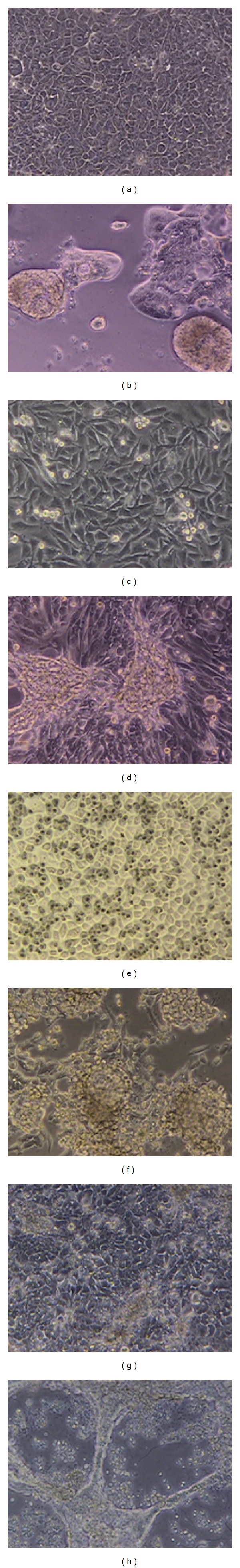
Morphology of MCF-10A, MB-231, PC-3, and P19 grown under 2D and 3D culture conditions, 5 to 6 days after establishment of the cultures. MCF-10A, an immortalized nontumorigenic breast epithelial cell line, shows epithelial morphology in 2D cultures (a), while these cells form adenoid-like, compact aggregates on the surface of the Matrigel matrix in 3D cultures without evidence of cells invading the matrix (b). MB-231 invasive breast cancer cells show bi- and tripolar morphology in 2D cultures (c). MB-231 cells growing under 3D culture conditions form monolayers and loose cell aggregates on the surface of the matrix. From these aggregates, individual cells invade the Matrigel matrix (d). PC-3 prostate cancer cells show epithelial morphology in 2D cultures (e). When placed on Matrigel surface, PC-3 cells form aggregates, from which individual cells invade into the matrix, similarly to but less extensively as MB-231 cells (f). Embryonal teratocarcinoma-derived P19 cells also have epithelial morphology in conventional 2D cultures (g), while under 3D culture conditions cells form tube-like structures reminiscent of vasculogenic mimicry patterns (h). Magnification: 100x.

**Figure 2:**
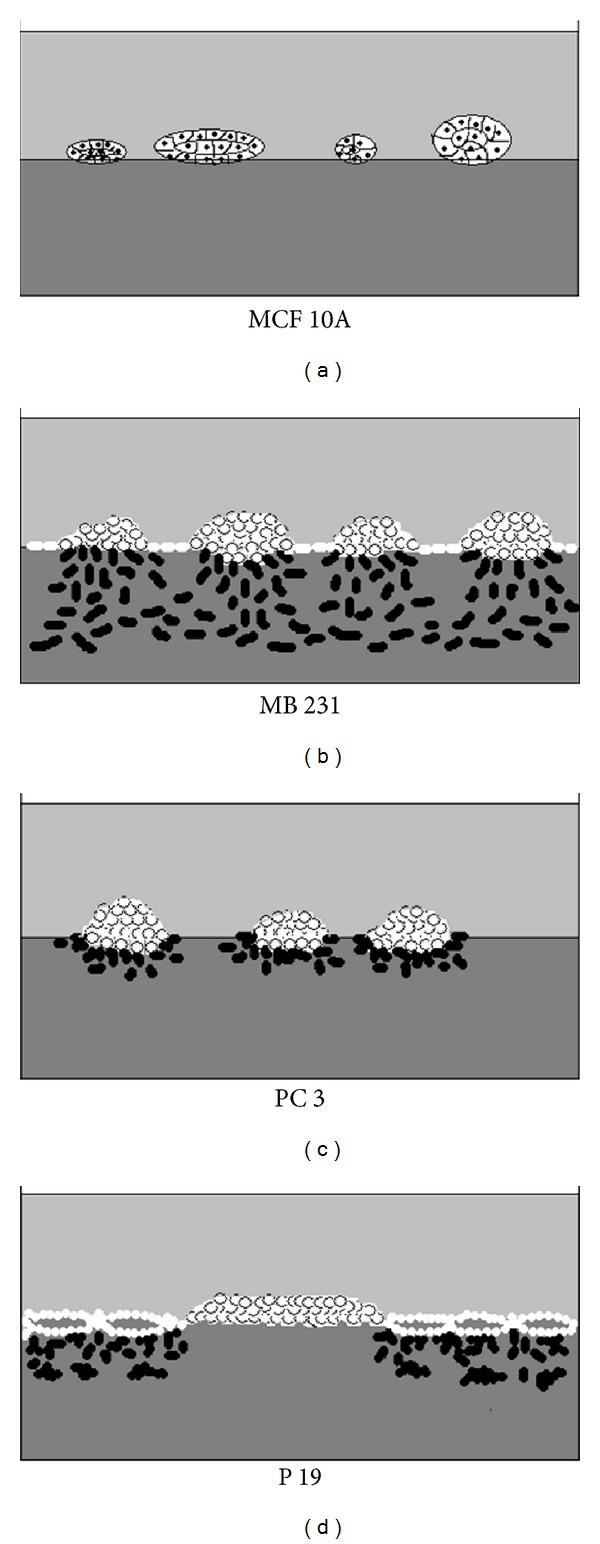
Schematic models of MCF-10A, MB-231, PC-3, and P19 cells growing under 3D culture conditions. MCF-10A cells form compact aggregates on the surface of the matrix without invading cells. MB-231 cells form monolayers and loose aggregates on the Matrigel surface and individual tumor cells invade the matrix from the surface as the cultures age. Similarly to MB-231 cells, PC-3 cells form aggregates on the matrix-surface and individual cells migrate into the gel as cultures age although matrix invasion is slower. P19 cells form tube-like structures reminiscent of vasculogenic mimicry patterns on the surface of the matrix, and later individual tumor cells invade the matrix in the proximity of vasculogenic mimicry patterns.

MCF-10A, an immortalized nontumorigenic breast epithelial cell line, showed epithelial morphology in 2D cultures (Figure 1(a)) and formed compact adenoid-like aggregates on the top of Matrigel in 3D cultures [44] (Figures 1(b) and 2). With time of culturing, the diameter of these aggregates was increasing, indicating viability of these cells under 3D culture conditions. In addition to the cell aggregates, MCF-10A cells also formed a discontinuous monolayer on the Matrigel surface but cells never invaded the matrix.

MB-231, an invasive breast cancer cell line, showed bi- and tripolar cell morphology in conventional 2D cultures (Figure 1(c)). In 3D cultures, MB-231 cells formed less compact aggregates than MCF-10A on the Matrigel surface (Figure 2(b)). From these aggregates elongated, bipolar cells migrated onto the surface, where the cells grew in monolayers. Another population of elongated cells invaded the matrix as individual cells (Figures 1(d) and 2(b)).

PC-3 prostate carcinoma cells had epithelial morphology in 2D cultures (Figure 1(e)). After seeding onto Matrigel, PC-3 cells formed aggregates first on the top of the matrix (Figure 1(f)). The aggregate-forming cells were mostly rounded in contrast to MB-231 cells, where the aggregate-composing cells had more elongated shape. During further culturing, some individual, bipolar cells invaded the matrix from PC-3 aggregates (Figures 1(f) and 2(c)). PC-3 cell invasion of Matrigel was slower and less extensive than that detected with MB-231 cells. On the surface of Matrigel, cells with epithelial morphology also formed a discontinuous monolayer (Figure 1(f)).

P19 mouse embryonal teratocarcinoma cells also showed epithelial morphology cultured under 2D culture conditions (Figure 1(g)). After seeding onto Matrigel matrix, P19 cells started to grow on the matrix surface in a single layer and formed tube-like structures that surrounded round matrix surfaces free from cells, similar to vasculogenic mimicry-formation of uveal melanoma cells in 3D cultures [18, 36] (Figure 1(d)). Beside tube (vasculogenic mimicry) formation, some cell aggregates were also formed on the matrix surface and individual cells invaded the Matrigel matrix (Figure 2(d)). Interestingly, these invasive cells were observed only under the tube-forming cells (Figure 2(d)). These observations indicated that the studied cell lines formed cell line-specific structures in 3D cultures and that only malignant tumor cells could invade the Matrigel matrix.

### 3.2. Tumor Cells Are More Resistant to HSV-1 under 3D Conditions Than in 2D Cultures

To examine the effect of ECM on HSV-1 infection, 2D and 3D cultures of MCF-10A, MB-231, PC-3, and P19 cells were inoculated by HSV-1 K26GFP by placing virus solutions on the surface of the cultures. HSV-1 K26GFP exhibits fluorescence upon replication [40]. Cultures were followed for evidence of cytopathic effects and virus replication (GFP expression/fluorescence) by an inverted fluorescence microscope.

**Figure 3:**
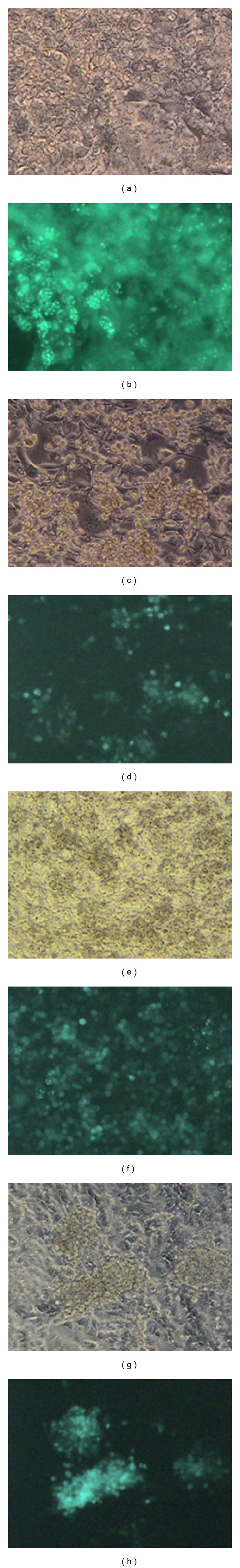
Morphology (a, c, e, and g) and GFP expression indicative of HSV-1 infection (b, d, f, and h) in two-dimensional MCF-10A, MB-231, PC-3, and P19 cultures 2 to 3 days after HSV-1 K26GFP inoculation. Wide spread GFP expression consistent with extensive virus replication was detected in MCF-10A (b) and PC-3 cultures (f). Moderate and low numbers of GFP-expressing cells were detected in MB-231 (d) and P19 cells (h), respectively. Magnification: 100x.

**Figure 4:**
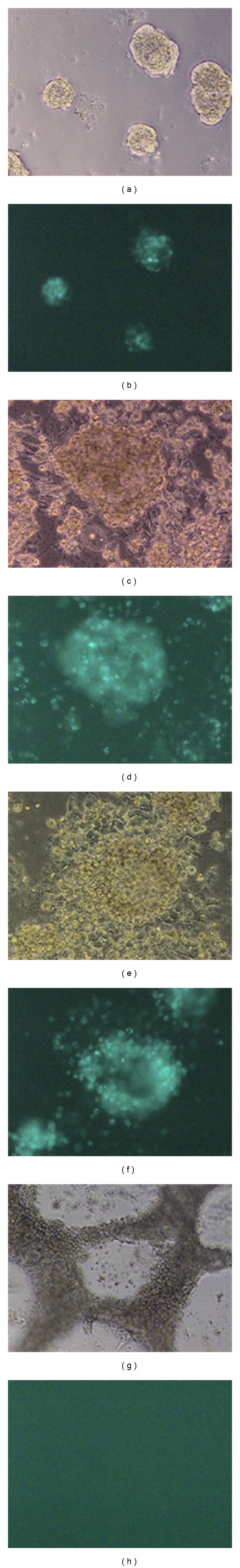
Morphology (a, c, e, and g) and GFP expression indicative of HSV-1 infection (b, d, f, and h) in three-dimensional MCF-10A, MB-231, PC-3, and P19 cultures 2 to 3 days after HSV-1 K26GFP inoculation. Most of MCF-10A cell aggregates on the Matrigel surface emitted green fluorescent light (b) consistent with extensive HSV-1 replication. Most of MB-231 cell aggregates on the Matrigel surface also expressed GFP (d) consistent with extensive HSV-1 replication. However, MB-231 cells invading the Matrigel matrix were often negative (d). Similarly, PC-3 cell aggregates on the matrix surface were extensively GFP-positive, while many invading cells were negative (f). Three-dimensional P19 cultures showed no to very limited GFP expression (h). Magnification: 100x.

Nontumorigenic breast MCF-10A cells showed high sensitivity to HSV-1 infection: both 2D and 3D cultures were destroyed by 3 days after the virus inoculation (Table 1). Cell destruction was associated with extensive GFP expression by virus (Figures 3(b) and 4(b)) consistent with virus replication in tumor cells.

The invasive MB-231 cell line was less sensitive to the HSV-1. Destruction of cells in 2D cultures was complete by the 6th day after virus inoculation, and 95% destruction of tumor cells was observed in 3D cultures only at the 8th day after virus inoculation (Table 1). In 3D cultures, a low number of viable tumor cells, most of which were invading the Matrigel matrix as individual cells, remained detectable at the end of the 14-day observation period. Cell destruction in 2D and 3D cultures was associated with GFP expression by virus (Figures 3(d) and 4(d)) consistent with virus replication in tumor cells.

HSV-1 inoculation of PC-3 cells showed outcomes similar to MB-231 cells. In 2D cultures, 100% of PC-3 cells were destroyed by 6 days following virus inoculation (Table 1). In 3D cultures, 95% of tumor cells were destroyed by 9 days following virus inoculation (Table 1). Cell destruction in 2D and 3D cultures was associated with GFP expression by virus (Figures 3(f) and 4(f)) consistent with virus replication in tumor cells.

Among the studied cell lines, P19 mouse embryonal teratocarcinoma cells were the most resistant to HSV-1. HSV-1-mediated destruction of 2D cultures was complete after 7 days following virus inoculation (Table 1). In 3D cultures, HSV-1-mediated destruction of tumor cells never reached 95% during the 2-week observation period (Table 1). Cell destruction in 2D was associated with GFP expression by virus (Figure 3(h)). In 3D cultures, tumor cell destruction was associated with GFP expression for several days following virus inoculation. However, GFP expression and cell destruction dramatically decreased during the second week of virus infection with no detectable GFP expression remaining after 14 days of virus inoculation (Figure 4(h)).

### 3.3. ECM Exposure Inhibits HSV-1 Replication after Virus Entry into Tumor Cells

To determine whether exposure to the ECM (Matrigel) can inhibit HSV-1 replication after virus entry into tumor cells in 3D cultures, 2D monolayers of MCF-10A, MB-231, PC-3, and P19 cells were inoculated with HSV-1 K26GFP and incubated with the virus for 1 hour allowing virus entry into the tumor cells. Cells were then scraped off and were divided into equal parts that were used to initiate 2D or 3D cultures. For 2D cultures, HSV-1 infected cells were mixed with culture media and were plated on culture dishes. For 3D cultures, HSV-1 infected cells were mixed with Matrigel-containing media and were plated on culture dishes. At 18 hours after the establishment of 2D and 3D cultures, cultures were examined under an inverted fluorescence microscope for evidence of GFP expression (viral replication) and the percentage of GFP-expressing cells among all tumor cells was determined. The 18-hour incubation period was selected to allow events of the viral replication cycle to take place in infected cells but not to provide enough time for spread of virus and replication in a new set of cells following the first replication cycle. These experiments were performed at two different multiplicities of infection (MOI): 0.01 and 1 plaque forming units (PFUs/cell). For control cultures, cells incubated with sterile PBS (mock infection) for 1 hour were scraped off and used for the establishment of 2D and 3D cultures. Mock infected control cultures never expressed GFP (data not shown).

As documented in Table 2, when nontumorigenic MCF-10A breast cells previously inoculated with HSV1 K26GFP at MOI of 1 were used for the establishment of 2D and 3D cultures, GFP expression (viral replication) was detected in 90% of cells in 2D cultures and 80% of cells in 3D cultures. In the case of invasive MB-231 breast cancer cells previously inoculated with HSV1 K26GFP at MOI of 1, GFP expression (viral replication) was detected in 80% of cells in 2D cultures and 60% of cells in 3D cultures 18 hours following the establishment of the 2D and 3D cultures (Table 2). In the case of PC-3 prostate cancer cells previously inoculated with HSV1 K26GFP at MOI of 1, GFP expression (viral replication) was detected in 70% of cells in 2D cultures and 50% of cells in 3D cultures 18 hours following the establishment of the 2D and 3D cultures (Table 1). A similar trend was noted with P19 embryonal teratocarcinoma cells: when P19 cells that were previously inoculated with HSV1 K26GFP at MOI of 1 were used for the establishment of 2D and 3D cultures, GFP expression (viral replication) was detected in 40% of cells in 2D cultures and only 30% of cells in 3D cultures (Table 2).

Differences between the permissiveness of 2D and 3D cultures were even more pronounced when a lower (0.01 PFU/cell) multiplicity of infection was used with fivefold to tenfold reduction in the percentage of tumor cells expressing GFP following exposure to the ECM in 3D cultures (Table 2). These findings indicate that exposure of tumor cells to the ECM (Matrigel) can inhibit HSV-1 replication after virus entry.

We have shown in previous studies that uveal melanoma cells grown in ECM-containing 3D cultures have increased resistance against HSV-1-mediated destruction when compared to cells cultured without ECM (Matrigel) [18, 36]. Using four cell lines including MCF10A immortalized nontumorigenic breast epithelial cells, MB231 human breast cancer cells, PC3 human prostate cancer cells, and P19 mouse embryonal carcinoma cells, we show in this current study that tumor cell lines other than melanoma are also more resistant to HSV-1-mediated destruction in 3D cultures relative to cells grown in 2D.

It is well known that cancer cells grown in 3D cultures are more resistant to chemotherapeutic agents and radiation than cells cultured under 2D conditions [27–29]. Consequently, 3D tumor cell cultures have been widely used to study tumor resistance mechanisms against chemotherapeutic agents and radiation [27–29]. Our observations reported here suggest that cancer cells grown in 3D cultures can also provide useful experimental platforms to study tumor resistance mechanisms against HSV-1 oncolytic therapy.

Earlier we have shown that the Matrigel matrix can form a physical barrier that can decrease or inhibit virus spread in 3D tumor cell cultures [18]. In our current study, tumor cell types that were capable of invading Matrigel demonstrated increased resistance against HSV-1-mediated destruction in 3D cultures, while MCF10A immortalized nontumorigenic breast epithelial cells that could not invade Matrigel in 3D cultures were equally sensitive to HSV-1-mediated destruction under 2D and 3D conditions. These findings could suggest that tumor cells that were invading Matrigel in 3D cultures were shielded from HSV-1 and that this mechanical barrier function of Matrigel was the only reason why 3D tumor cultures had increased resistance against destruction by virus. However, previous observations in our laboratory [18] and several lines of observations in the current study also suggested that the increased resistance of tumor cells against HSV-1 in 3D cultures can also be due to additional mechanisms including ECM-mediated inhibition of HSV-1 replication following virus entry into the cells. In experiments that involved the placement of tumor cells on the surface of Matrigel and waiting for tumor cells to invade Matrigel for the establishment of 3D tumor cultures before placement of HSV-1 inocula on the top of the 3D cultures, virus infection (GFP expression) was commonly detected in tumor cells that have invaded Matrigel suggesting that HSV-1 could reach tumor cells through the ECM. Furthermore, in experiments where previously HSV-1-infected tumor cells were surrounded with ECM, virus replication (GFP expression) was significantly reduced relative to controls. Interestingly, our observations indicated that at a higher multiplicity of virus inoculation (MOI), ECM exposure was not as efficient in the inhibition of HSV-1 replication in tumor cells following cell entry than at lower MOIs.

It is well documented that adhesion of cancer cells to the ECM mediates drug- and radiation-resistance [23, 28, 45]. The connection of tumor cells to ECM proteins such as collagen and laminin through cell adhesion molecules is associated with tumor cell survival and drug resistance through the activation of a variety of pathways [46–48]. It is possible that ECM-mediated signalling also affects viral replication through some of these pathways but it is clear that the exact mechanisms of ECM-mediated inhibition of HSV-1 replication in 3D cultures will need to be defined in future experiments.

## 4. Conclusions

Observations reported here confirm and extend previous observations related to the increased resistance of ECM-containing 3D tumor cell cultures against HSV-1 mediated destruction relative to traditional 2D monolayer cultures. We also showed here that one mechanism responsible for the increased resistance of tumor cells to HSV-1 infection in 3D cultures is an ECM-mediated inhibition of virus replication following virus entry into the cells. Observations reported here suggest that 3D cancer cell cultures can provide useful experimental platforms to identify and study novel tumor resistance mechanisms against HSV-1 oncolytic therapy and thus may lead to improved strategies of oncolytic virotherapy.

## Figures and Tables

**Table 1:** Elapsed time from inoculation of HSV-1 K26GFP (at MOI = 0.5 PFU per cell) to at least 95% destruction of 2D, and 3D cultures of MCF-10A, MB-231, PC-3, and P19 cells.

Cell type	Culture	95% destruction (day after virus inoculation)
MCF-10A	2D	3
	3D	3

MB-231	2D	6
	3D	8

PC-3	2D	6
	3D	9

P19	2D	7
	3D	N/A

**Table 2:** HSV-1 replication (GFP expression) in MCF-10A, MB-231, PC-3, and P19 cells first exposed to HSV-1 K26GFP for 1 hour under 2D conditions to ensure exposure of cells to virus and then cultured for 1 day either under 2D or 3D conditions.

Cell type	Culture type	% of cells GFP-positive at day 1*	
		MOI = 0.01	MOI = 1
MCF-10A	2D	ND	90
	3D	ND	80

MB-231	2D	0.5	80
	3D	0.1	60

PC-3	2D	2	70
	3D	0.4	50

P19	2D	0.2	40
	3D	0.02	30

*Percentage of GFP-expressing cells was determined by counting the number of GFP-expressing and GFP-negative cells in 16 high power microscopic fields for each studied type of treatment at two different multiplicities of infection (MOI = 0.01 and 1 PFU per cell). ND: not done.
